# Factors affecting postoperative blood loss in children undergoing cardiac surgery

**DOI:** 10.1186/1749-8090-9-32

**Published:** 2014-02-11

**Authors:** David Faraoni, Philippe Van der Linden

**Affiliations:** 1 Department of Anesthesiology, Centre Hospitalier Universitaire (CHU) Brugmann - Queen Fabiola Children’s University Hospital (QFCUH), Free University of Brussels, 15 JJ Crocq Av. 1020, Brussels, Belgium

**Keywords:** Congenital heart surgery, Bleeding management, Age, Point-of-care monitoring

## Abstract

We hypothesized that the influence of cyanotic disease on postoperative blood loss is closely related to age in children undergoing cardiac surgery. Here, we demonstrate that the presence of a cyanotic disease is associated with increased postoperative blood loss in children aged 1 to 6 months. Children with cyanotic disease and aged < 1 month who received fresh frozen plasma during cardiopulmonary bypass had less postoperative blood loss and higher maximal clot firmness on FIBTEM than cyanotic children from all other groups. Additional studies are needed to define optimal pathophysiology-based management in children undergoing cardiac surgery.

## Introduction

Children who undergo cardiac surgery develop postoperative coagulopathy due to hemodilution and surgical and patient-related factors [1,2]. Age and the presence of cyanotic disease are well recognized as predisposing factors for postoperative coagulopathy [3,4]. The effects of these two factors on ROTEM® have been investigated in different studies [5,6], but the interaction between these factors and the clinical manifestation of postoperative bleeding remains to be defined. We hypothesized that the influence of cyanotic disease on postoperative blood loss is closely related to age in children undergoing cardiac surgery.

## Methods

After approval by the local ethic committee (QFCUH Ethic Board), we retrospectively reviewed data from our departmental database for all children scheduled for elective cardiac surgery with cardiopulmonary bypass (CPB). The need for informed consent was waived due to the retrospective nature of the analysis. The anesthetic, CPB, and surgical techniques are standardized in our department. Tranexamic acid was systematically administered using the same dosing scheme. In terms of anticoagulation, unfractionated heparin (UFH, 4 mg/kg) was administered before aortic cannulation. The activated clotting time (ACT) (ACTII monitor, Medtronic BV, Kerkrade, the Netherlands) was used to guide additional UFH administration, maintaining a target ACT > 480 sec. At the end of CPB, protamine was administered to antagonize heparin activity. Adequate reversal was controlled using the ACTII monitor to comparing the ACT measured in the cartridge with and without heparinase (Medtronic BV, Kerkrade, the Netherlands).

In children older than 1 month of age, 6% hydroxyethyl starch (HES 130/0.4, Voluven®, Fresenius-Kabi Gmbh, Bad Homburg, Germany) was systematically used for CPB priming. In children < 1 month old, fresh frozen plasma (FFP) replaced the colloid solution. The transfusion algorithm was standardized as described previously [7]. Ten minutes after protamine administration, blood samples were drawn to measure platelet count, fibrinogen plasma concentration (Claus method), and parameters determined by the EXTEM and FIBTEM tests on ROTEM® (ROTEM®, TEM® International GmbH, Munich, Germany).

### Statistical analysis

Children were characterized into five groups according to age (< 1 month, 1-6 months, 6-12 months, 1-3 years, > 3 years). In each group, children with cyanotic and non-cyanotic disease were differentiated. Continuous variables were tested for normality using the Kolmogorov-Smirnov test. Data are presented as medians and interquartile ranges (25th percentile to 75th percentile). Categorical variables are expressed as number and fraction (%). Blood loss was measured in the 6th postoperative hour and expressed as a percentage of the child’s estimated blood volume (EBV). The EBV and coagulation parameters obtained after heparin antagonization were analyzed using a two-way ANOVA analysis of variance testing for a difference between age groups, a difference between cyanotic and non-cyanotic disease, and for the interaction between age and cyanotic disease. If significant, Bonferroni’s multiple comparison test was performed.

Statistical analyses were performed using Prism 6 for Mac OS (version 6.0a; GraphPad Software Inc., San Diego, California, USA, http://www.graphpad.com). A p-value < 0.05 was considered significant.

### Findings

Data was obtained from 182 patients (Table 1). Postoperative blood loss was significantly influenced by age (p = 0.003) and the presence of a cyanotic disease (p = 0.01). We also identified a significant interaction between these two factors (p = 0.03).

**Table 1:** Demographic characteristics of the population

**Age**	**Cyano.**	**N**	**Age (mo.)**	**Male (%)**	**RACHS**	**Height (cm)**	**Weight (kg)**
< 1 mo.	1	8	0.3 (0.1 to 0.3)	6 (75)	3 (3 to 4)	50 (46 to 51)	3.3 (2.9 to 4.0)
	0	21	0.3 (0.2 to 0.7)	12 (57)	4 (3 to 4)	48 (46 to 50)	2.9 (2.7 to 3.7)
1 to 6 mo.	1	21	3.8 (3.0 to 5.0)	12 (57)	3 (2 to 4)	57 (55 to 60)	4.4 (3.7 to 6.0)
	0	24	2.4 (1.7 to 3.7)	10 (42)	2 (2 to 3)	55 (51 to 58)	4.4 (3.6 to 5.6)
6 to 12 mo.	1	10	7.0 (6.6 to 8.2)	6 (60)	2 (2 to 3)	67 (64 to 70)	6.6 (5.9 to 8.0)
	0	15	9.0 (8.0 to 9.5)	8 (53)	2 (2 to 3)	68 (65 to 71)	7.0 (5.4 to 7.5)
1 to 3 yr.	1	13	15.7 (13.2 to 18.9)	9 (69)	2 (2 to 3)	76 (71 to 85)	9.9 (7.6 to 12.1)
	0	25	18.6 (15.0 to 26.8)	12 (48)	2 (2 to 3)	78 (69 to 87)	9.8 (6.6 to 11.2)
> 3 yr.	1	12	46.9 (38.0 to 67.3)	8 (67)	3 (2 to 3)	100 (92 to 104)	14.1 (13.3 to 15.9)
	0	33	73.0 (47.4 to 120.0)	17 (52)	3 (2 to 3)	113 (104 to 134)	21.0 (15.6 to 28.7)

Data are expressed as median and interquartile range (25th percentile to 75th percentile). Cyano, cyanogen; RACHS, Risk Adjustment for Congenital Heart Surgery.

**Figure 1:**
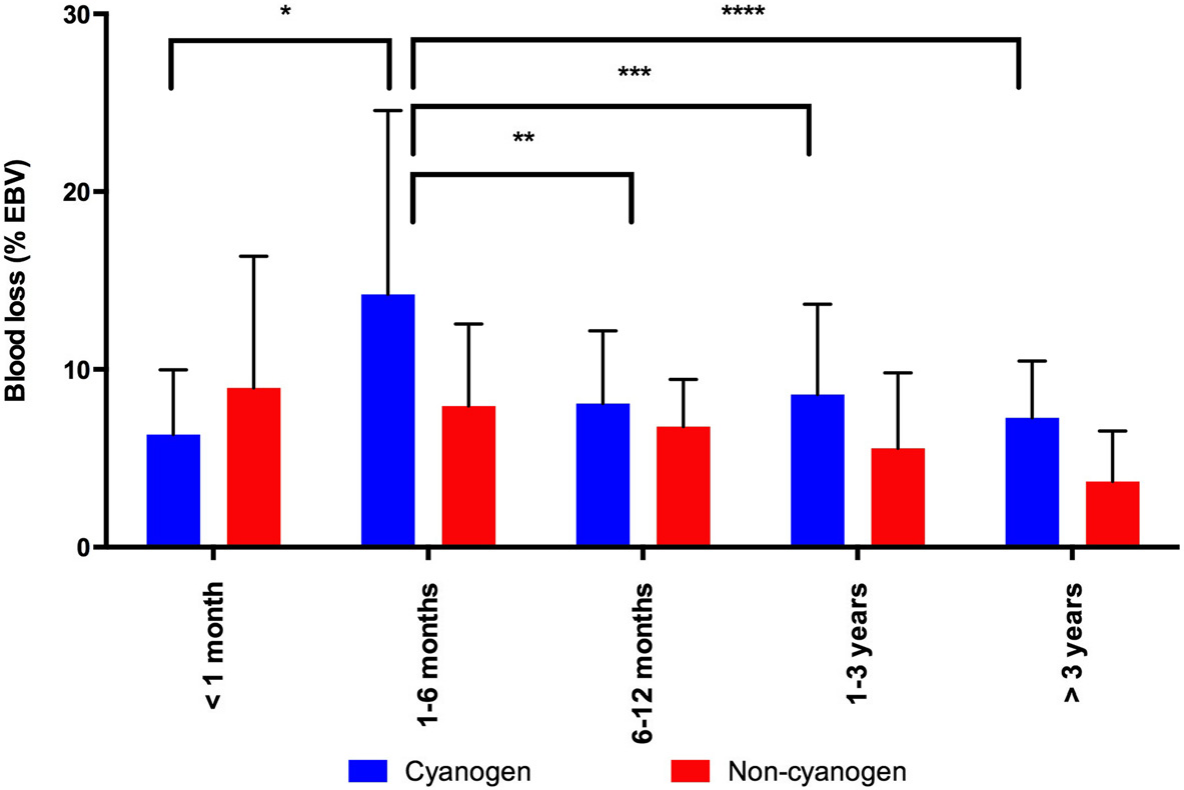
**Blood loss based on age and the presence of cyanotic disease.** *p < 0.005 comparing < 1 month and 1-6 months with cyanotic disease (mean difference between groups 7.9% estimated blood volume (EBV), 95% CI 1.4 to 14); ** p < 0.05 comparing 1-6 months and 6-12 months with cyanotic disease (mean difference between groups -6.1% EBV, 95% CI -12 to -0.15); *** p < 0.05 comparing 1-6 months and 1-3 years with cyanotic disease (mean difference between groups -5.6% EBV, 95% CI -11 to -0.12); **** p < 0.005 comparing 1-6 months and > 3 years with cyanotic disease (mean difference between groups -6.9% EBV, 95% CI -13 to -1.3).

Postoperative bleeding was significantly higher in the cyanotic population compared to the non-cyanotic population, but only in children aged 1 to 6 months. In the non-cyanotic population, postoperative blood loss was not influenced by age (Figure 1). Regarding FFP and platelet transfusion, no difference was observed between the cyanotic and non-cyanotic populations in the different age groups (Table 2).

**Table 2:** Transfusion data

**Age group**	**Cyano.**	**N**	**Expo. to RBC (%)**	**RBC in CPB (%)**	**Expo. to FFP (%)**	**FFP in CPB (%)**	**Expo. to PLT (%)**
< 1 mo.	1	8	6 (75)	4 (50)	2 (25)	8 (100)	0 (0)
	0	21	19 (90)	18 (86)	12 (57)	21 (100)	5 (24)
1 to 6 mo.	1	21	15 (71)	8 (38)	2 (9)	2 (9)	1 (5)
	0	24	19 (79)	21 (87)	1 (4)	0 (0)	1 (4)
6 to 12 mo.	1	10	2 (20)	1 (10)	0 (0)	0 (0)	0 (0)
	0	15	11 (73)	5 (33)	0 (0)	1 (7)	0 (0)
1 to 3 yr.	1	13	4 (31)	2 (15)	1 (8)	1 (8)	0 (0)
	0	25	13 (52)	4 (16)	0 (0)	1 (4)	0 (0)
> 3 yr.	1	12	0 (0)	0 (0)	0 (0)	0 (0)	0 (0)
	0	33	1 (3)	1 (3)	0 (0)	0 (0)	0 (0)

Data are expressed number and percentage. Cyano, cyanogen; Expo, exposition; RBC, red blood cell; FFP, fresh frozen plasma; PLT, platelets; CPB, cardiopulmonary bypass.

**Figure 2:**
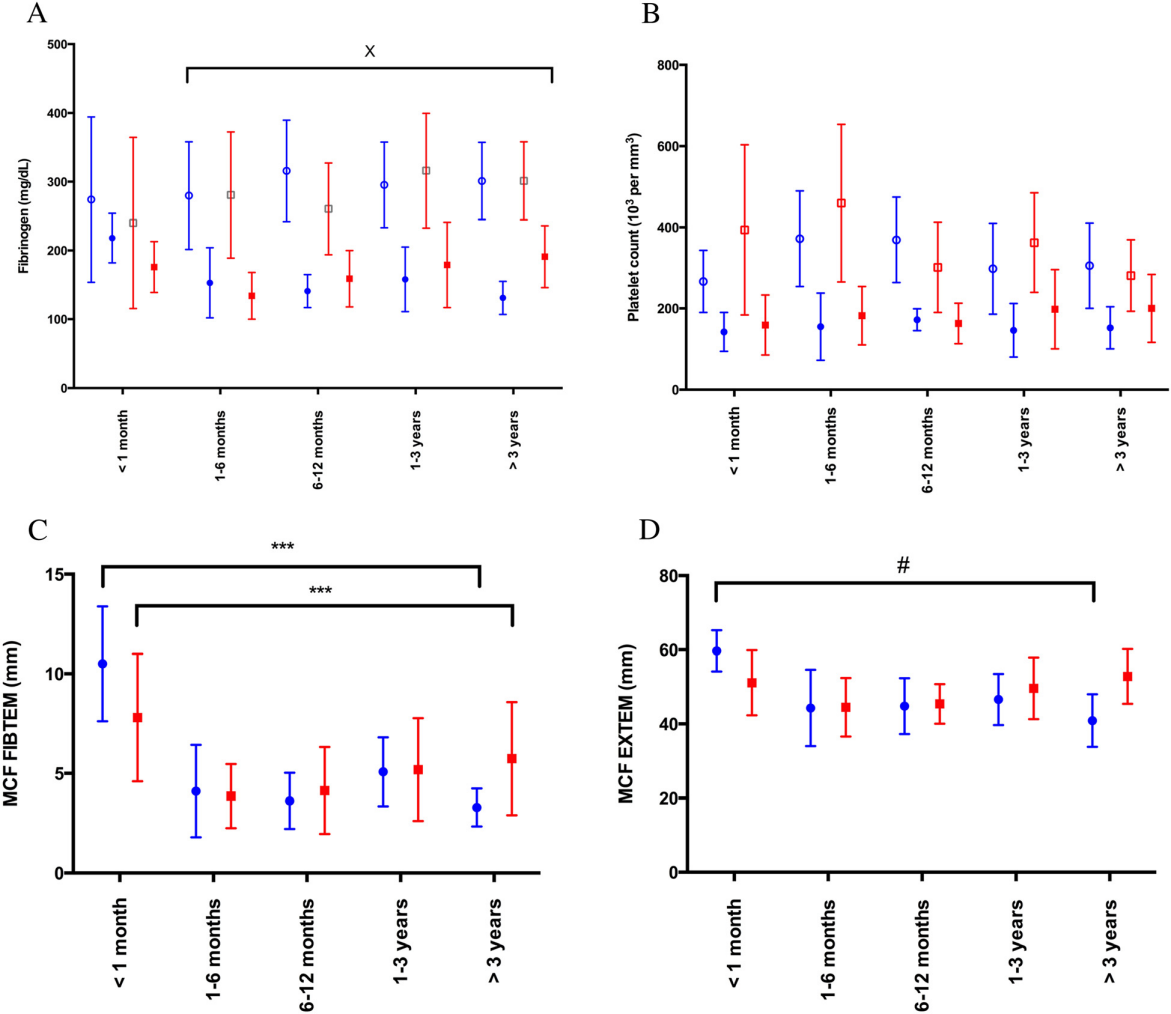
**Coagulation parameters based on age and the presence of cyanotic disease. (A)** Fibrinogen levels measured using the Clauss method at baseline and after cardiopulmonary bypass (CPB) (p < 0.001). **(B)** Platelet count at baseline and after CPB (p < 0.001). **(C)** Maximal clot firmness obtained on FIBTEM after CPB. **(D)** Maximal clot firmness obtained on EXTEM after CPB. ***p < 0.001 comparing < 1 month and all other groups with cyanotic and non-cyanotic diseases, #p < 0.01 comparing < 1 month and all other groups with cyanotic diseases. Blue, cyanotic patients; red, non-cyanotic patients; clear symbols represent baseline tests; and full symbols represent post-CPB tests.

Fibrinogen levels were significantly influenced by age (p < 0.001) but not by the presence of cyanotic disease (p = 0.3). Interestingly, baseline fibrinogen levels were significantly different from post-CPB levels in all age groups (p < 0.001) except in children < 1 month of age (Figure 2A). In contrast, platelet count was not influenced by age (p = 0.7), but it was influenced by the presence of cyanotic disease (p = 0.03; Figure 2B). Similar to fibrinogen, baseline and post-CPB platelet counts were significantly different (p < 0.001). Maximal clot firmness (MCF) obtained on EXTEM (p < 0.001) or FIBTEM (p < 0.001) were the only ROTEM® parameters influenced by age. The MCF obtained on FIBTEM was significantly higher in the presence of cyanotic or non-cyanotic disease in children aged < 1 month compared to all other age groups (p < 0.001 for all comparisons). The same difference in MCF was observed with EXTEM in children with cyanotic disease (p < 0.01 for all comparisons) but not in children with non-cyanotic disease (p > 0.05 for all comparisons). Finally, ROTEM® parameters were not significantly different between cyanotic and non-cyanotic children (Figure 2C and 2D).

## Discussion

Based on our preliminary descriptive analyses, postoperative blood loss was significantly influenced by age in the cyanotic, but not the non-cyanotic, population. The addition of FFP during CPB in children < 1 month of age increased the MCF on FIBTEM and EXTEM in the presence of cyanotic disease. The addition of FFP to CPB in children < 1 month of age also decreased postoperative blood loss in children with cyanotic disease, but the evidence seems to be weak in the non-cyanotic population.

Bleeding disorders and abnormal hemostasis in children with cyanotic disease are well known [8]. Coagulation defects, such as thrombocytopenia, and factor deficiencies, including low fibrinogen levels and impaired fibrinogen polymerization, have also been described [9]. In a recent study, Jensen et al. reported that children with cyanotic disease are in a hypocoagulable state related primarily to impaired fibrinogen function, whereas thrombocytopenia, if present, is not associated with severe platelet dysfunction [10]. On the other hand, data regarding post-CPB platelet dysfunction are sparse [11,12]. Using a point-of-care assessment of platelet aggregation in children undergoing cardiac surgery, Hofer et al. recently reported that cyanotic patients exhibit better platelet aggregation than non-cyanotic patients despite increased bleeding tendency, which could be explained by the predominant fibrinogen dysfunction in children with cyanotic disease [11]. However, the relationship between platelet function and perioperative blood loss has only been studied in a preliminary study; Ranucci et al. observed that the use of platelet function assays is not supported in the pediatric cardiac population [12]. In addition, no study has assessed platelet and fibrinogen function simultaneously in children undergoing cardiac surgery.

In our population, we observed that age influences postoperative blood loss only in children with cyanotic disease until the age of 6 months, but no difference was found between age groups in the non-cyanotic population. Further well-designed prospective studies are needed to better understand the pathophysiological mechanisms, but we hypothesize that the immature coagulation system, especially fibrinogen function, could be more impaired in children < 6 months of age in the presence of cyanotic disease. After 6 months of age, the potential difference in hemostatic physiology between cyanotic and non-cyanotic diseases is no longer associated with clinically relevant differences.

We also observed that the addition of FFP significantly decreased postoperative blood loss in children aged < 1 month compared to those aged 1 to 6 months. The decrease in postoperative blood loss seems to be particularly relevant in children with cyanotic disease. From a pathophysiological point-of-view, the effect of FFP on blood loss could be explained by the supplementation of clotting factors, especially fibrinogen. This conclusion is supported by MCF on FIBTEM being the only ROTEM® parameter increased in children aged < 1 month compared to other age groups. In addition, we observed that hemodilution obtained during CPB significantly decreased the platelet count in all children, whereas the fibrinogen level significantly decreases in children aged > 1 month. This observation could support our hypothesis that FFP helped maintain the fibrinogen level when used in children aged < 1 month. If the fibrinogen level needs to be maintained, cryoprecipitate or fibrinogen concentrates could also be considered and could results in a lower degree of hemodilution [13].

A recent study reported that fibrinogen concentrate should be used as a first line therapy in bleeding patients after CPB [14]. If no data exist in the pediatric population, further prospective trials can be performed to assess the efficacy of fibrinogen concentrates compared to FFP in children undergoing cardiac surgery. This concentrate-based approach could decrease hemodilution and decrease the need for transfusion. The results of these analyses should be interpreted with caution due to the risk of bias, but our hypothesis can be assessed in a large, prospective trial.

## Conclusion

In conclusion, our understanding of the pathophysiological mechanisms leading to increases bleeding risks in the pediatric population, particularly cyanotic children, is weak. Further prospective studies are needed to assess the relationship between postoperative blood loss, hemodilution, platelet dysfunction, and/or fibrinogen deficiency. A better understanding of these mechanisms would lead to better goal-directed management, considering that children with cyanotic disease may need to be treated with different targets than those with non-cyanotic disease.

## Abbreviations

CPB: Cardiopulmonary bypass; ACT: Activated clotting time; FFP: Fresh frozen plasma; EBV: Estimated blood volume; MCF: Maximal clot firmness.

## Competing interests

The authors declare that they have no competing interests.

## Authors’ contributions

DF analyzed the data, wrote the manuscript. PVdL helped write the manuscript. Both author read and approved the final manuscript.
